# Inactivated SARS-CoV-2 Exposure Induces Inflammation and Impairs Reproductive Outcomes in Mice

**DOI:** 10.3390/v18101112

**Published:** 2026-10-08

**Authors:** Patrícia Ribeiro de Carvalho França, João Pedro Barros de Paiva, Amanda Gabriel Valente, Alan Silva Minho, Renato Sampaio Carvalho, Rosangela Ribeiro de Carvalho, Luiz Ricardo Berbert, Marcel Frajblat, Maria Marta Figueiredo, Daisymara Priscila de Almeida Marques, Jordana Grazziela Alves Coelho-dos-Reis, Patricia Dias Fernandes

**Affiliations:** 1 Laboratório de Farmacologia da Dor e da Inflamação, Instituto de Ciências Biomédicas, Universidade Federal do Rio de Janeiro, Rio de Janeiro 21941-902, Brazil; patriciaribeiro.ufrj@yahoo.com.br (P.R.d.C.F.); jbarrosdepaiva@gmail.com (J.P.B.d.P.); amanda.valenteg@gmail.com (A.G.V.); a_minho@me.com (A.S.M.); renatosampaiocarvalho@yahoo.br (R.S.C.); 2 Laboratório de Toxicologia Ambiental, Departamento de Ciências Biológicas, Escola Nacional de Saúde Pública, Fiocruz, Rio de Janeiro 21041-210, Brazil; rosangelacarvalho@gmail.com; 3 Coordenação de Atividades com Modelos Biológicos Experimentais, Universidade Federal do Rio de Janeiro, Rio de Janeiro 21941-902, Brazilmarcel.frajblat@gmail.com (M.F.); 4 Departamento de Ciências Naturais da Terra, Universidade do Estado de Minas Gerais, Belo Horizonte 35501-170, Brazil; mariamartafigueiredo@gmail.com; 5 Departamento de Microbiologia, Instituto de Ciências Biomédicas, Laboratório de Virologia Básica e Aplicada, Universidade Federal de Minas Gerais, Belo Horizonte 31270-901, Brazilreisjordana@gmail.com (J.G.A.C.-d.-R.)

**Keywords:** SARS-CoV-2 virus, reproductive dysfunction, inflammation, pregnancy loss

## Abstract

COVID-19 has been associated with reproductive dysfunction, although the underlying mechanisms remain unclear. Here, we investigated the effects of inactivated SARS-CoV-2 (iSARS-CoV-2) exposure on reproductive outcomes in K18-hACE2 mice. Pregnant females exposed during early gestation showed reduced fetal number, increased resorptions and decreased placental efficiency. These alterations were associated with pulmonary inflammation, characterized by elevated TNF-α, IL-6 and IFN-γ levels and histopathological damage. In males, exposure impaired the sperm count, motility and reproductive performance, and negatively affected pregnancy outcomes in untreated females. Notably, inflammatory responses occurred in the absence of viral replication, indicating that viral components can trigger systemic effects. Together, these findings demonstrate that inactivated SARS-CoV-2 induces inflammation that compromises fertility and gestational success in both sexes.

## 1. Introduction

Severe acute respiratory syndrome caused by the SARS-CoV-2 virus was responsible for a pandemic that began in March 2020 and resulted in tragic consequences for global public health and the medical system, causing millions of deaths worldwide [1,2]. As a result of the rapid spread and high infectivity rate of Coronavirus Disease 2019 (COVID-19), researchers and pharmaceutical companies had to rapidly develop vaccines using new or pre-existing technologies, and, due to massive vaccination campaigns, it was possible to achieve control of the severity of cases and a reduction in the number of deaths [3].

The SARS-CoV-2 virus binds via its anchoring protein, the Spike protein, to the angiotensin-converting enzyme-2 (ACE-2) receptor, which is expressed on the membrane of many host cells, including placental cells, particularly in the syncytiotrophoblast [4]. Furthermore, co-expression of transmembrane serine protease 2 (TMPRSS2) in these cells promotes viral entry by stimulating the fusion of viral particles with the host cell membrane and related viral replication [5]. However, vertical transmission of COVID-19 from mother to fetus during pregnancy is rare due to the variability of ACE-2 receptor and TMPRSS-2 co-expression in each trimester [6,7,8].

Like other RNA viral infections in pregnancy, infection caused by the SARS-CoV-2 virus is associated with worrying maternal and perinatal outcomes [9,10,11], such as preeclampsia, poor maternal vascular perfusion, pre-term birth, intrauterine growth retardation, reduced birth weight and neuropsychomotor development, and even sudden fetal death [12,13]. Furthermore, even asymptomatic infections can cause premature aging and impaired uterine blood flow in the placenta, thus disrupting fetal growth [14]. In addition to the female reproductive tract, the ACE-2 receptor is also expressed in Leydig and Sertoli cells, as well as spermatogonia, suggesting that the testes may also be potential targets of SARS-CoV-2 infection [15].

SARS-CoV-2 infection can occur through several mechanisms, such as inflammatory responses, oxidative stress, and apoptotic pathways. Activation of these pathways can result in disruption of male reproductive functions [16] and overproduction of reactive oxygen species, which can accelerate activation of the NFκB–TLR (nuclear factor kappa-light chain-enhancer of activated B cells–toll-like receptor) pathways, culminating in the release of cytokines that further exacerbate the inflammatory response [17].

Some clinical data report orchitis (inflammation of the testicles) as one of the common symptoms during the symptomatic stage of COVID-19 [18,19]. Furthermore, post-mortem analyses of testicles from COVID-19 patients have revealed signs of testicular pathology, including tubular injury, depletion of germ cells and Leydig cells, and leukocyte infiltration; however, the presence of SARS-CoV-2 RNA in the testis and semen is a rare event [20].

In addition, changes in male fertility parameters, such as decreased testosterone levels and impaired sperm parameters, including reduced concentration and motility, are reported for months after recovery in all elderly COVID-19 patients, as well as men of reproductive age (20–49 years) [21].

Therefore, the objective of our work is to evaluate the impact of inflammation caused by inactivated SARS-CoV-2 virus (iSARS-CoV-2) on spermatogenesis and the reproductive performance of K18-hECA2 transgenic male mice, as well as to determine possible changes related to fetal and placental impairment of K18-hECA2 transgenic female mice during pregnancy.

## 2. Materials and Methods

### 2.1. Animal Model

B6.Cg-Tg(K18-ACE2)2Prlmn/J (K18-hACE2) transgenic hemizygous mice were used in this study. This strain was selected because spike–ACE2 engagement is biologically active independently of viral replication: exposure to spike protein alone downregulates ACE2, disrupts local renin–angiotensin homeostasis and induces inflammatory injury in vivo [22,23], and the same non-replicating spike-derived stimulus elicits a markedly stronger response in K18-hACE2 mice than in wild-type mice [22]. hACE2 expression has been documented in the female reproductive tract of this strain [24], and ACE2 is broadly expressed in gonadal tissue [25]. The model provides a receptor-relevant context for assessing inflammatory and reproductive outcomes of exposure to inactivated SARS-CoV-2 in the absence of productive infection. Male and female mice, aged 6–8 weeks and weighing approximately 20–25 g at the beginning of the experiments, were obtained from the Mice Central Facility of the Federal University of Rio de Janeiro. Only healthy animals without apparent clinical abnormalities were included in the study. Animals were randomly allocated to experimental groups, and no animals met exclusion criteria or were excluded from the analyses.

The animals were housed with free access to food and water under controlled environmental conditions (22 ± 2 °C; 12 h light/dark cycle) at the Animal Experimentation Unit of the Institute of Biomedical Sciences, Federal University of Rio de Janeiro (UFRJ). All experimental procedures were conducted in accordance with Brazilian National Council for the Control of Animal Experimentation (CONCEA) guidelines (Law No. 11.794/2008) and were approved by the Ethics Committee for the Use of Animals (CEUA) of the Center for Health Sciences, UFRJ (Protocol No. 93/22).

### 2.2. Obtaining the Inactivated SARS-CoV-2 Virus

The SARS-CoV-2 virus, WUHAN strain, was produced in the VERO CCL81 cell line at the titer of 1.25 × 10^6^ PFU/mL in a biosafety security level 3 lab at the Federal University of Minas Gerais, UFMG. The virus was inactivated by incubation with paraformaldehyde 1% solution for 1 h, followed by exposure to UV light for another hour. Inactivation was confirmed by plaque assay, as previously described by our group [26].

### 2.3. Mating and Assessment of Maternal Toxicity

Mating was performed by placing two females in a male’s cage during the last two hours of the dark cycle. After the presence of a vaginal plug or the presence of sperm in the vaginal wash, the females were separated and weighed. This day was considered day 0 of pregnancy. On days 5, 10, 15, and 18 of pregnancy (P5, P10, P15, and P18, respectively), the females were weighed to monitor their weight gain and general condition throughout pregnancy. On the 18th day, the females were weighed immediately before cesarean section; thus, P18 body weight included the gravid uterus and its contents. After cesarean section, the gravid uterus and its contents were removed and weighed separately. The maternal toxicity (MT) was calculated by the formula:

MT = (weight on day 18 before cesarean section − weight on day 0) − weight of the gravid uterus with all contents

### 2.4. Cesarean Section and Analysis of Types of Resorptions

On P18, cesarean sections were performed. The uterus was removed and weighed with its contents (placenta, fetus, and amniotic fluid). The uterine wall was carefully opened, and the number of live and dead fetuses and resorptions were counted. The Salewski method was used to identify all implantation sites, including those that were completely resorbed. Briefly, the opened uterus was immersed in a 10% ammonium sulfide solution for 10 min, rinsed in running water, and then immersed for another 10 min in a solution containing equal parts of 20% potassium ferrocyanide and 1% hydrochloric acid [27]. Reabsorptions were classified as follows:

- Early reabsorption: if an implantation site (visualized after staining the fresh uterus using the Salewski technique) does not correspond to a fetus or to remains of an embryo and placenta visible to the naked eye;
- Intermediate reabsorption: if parts of the body of embryos and placentas cannot be differentiated macroscopically;
- Late reabsorption: if parts of the embryo and placenta can be differentiated macroscopically.

### 2.5. Instillation of Inactivated Virus in Pregnant Females to Assess Pregnancy Loss and Placental Efficiency

To assess the impact of the virus during gestation, after mating, females presenting a vaginal plug were anesthetized with ketamine (90 mg/kg) and xylazine (10 mg/kg) and instilled every day with 5 μL/nostril of 1 × 10^5^ PFU of the iSARS-CoV-2 virus during the first 7 days of gestation (period of embryo implantation). On P18, a cesarean section was performed for analyses of maternal toxicity, the total number of reabsorptions, and the total number and weight of fetuses, as well as their respective placentas.

Placental efficiency (PE), an index of maternal–fetal adaptation, was calculated using the ratio fetal weight/weight of the respective placenta.

### 2.6. Instillation of Inactivated Virus in Males for Total Sperm Count and Classification of Sperm Motility

To assess whether the iSARS-CoV-2 virus could interfere with sperm production, male mice were anesthetized with ketamine (90 mg/kg) and xylazine (10 mg/kg) and instilled during two consecutive weeks with 5 μL/nostril of 1 × 10^5^ PFU of the iSARS-CoV-2 virus. On P15, mice were euthanized, and the testicles were removed, weighed and processed for histological analyses. The epididymis and vas deferens were placed in a specific buffer for gametes used for in vitro fertilization procedures (COOK Medical, Europe, Limerick, Ireland), and small cuts were made in the tail to facilitate sperm migration into the solution. Afterwards, a 20 μL aliquot was removed and placed in a Neubauer chamber for total counting and classification of sperm in relation to their motility as: A = progressive and fast sperm; B = progressive and slow sperm; C = motile sperm, but without progression; D = immobile sperm.

### 2.7. Morphological Analysis of Spermatozoa

To assess the percentage of morphologically abnormal spermatozoa (defects detected in the head or tail), a 10 μL aliquot of the suspension of the epididymis and vas deferens was removed, and a smear was prepared on a slide and carefully stained with the rapid panoptic kit (LaborClin, Rio de Janeiro, Brazil). Two hundred spermatozoa per animal were analyzed under an optical microscope at 1000× magnification, and normal or abnormal morphology was assessed following the criteria described in [28]. The presence or absence of defects in the head (e.g., double head, amorphous head, sharp hook, banana head, giant head, pinhead, round head) or tail (crooked flagellum, broken flagellum, coiled flagellum, short flagellum) of each spermatozoon was observed.

### 2.8. Analysis of Reproductive Performance in Males Instilled with the iSARS-CoV-2 Virus

In order to verify whether instillation with the iSARS-CoV-2 virus altered reproductive performance, i.e., the animals’ reproductive success rate, or whether it altered the number of implantation sites in nulliparous females, mice were anesthetized with ketamine (90 mg/kg) and xylazine (10 mg/kg) and instilled during 2 consecutive weeks with 5 μL/nostril of 1 × 10^5^ PFU of the iSARS-CoV-2 virus. The mating process was initiated for a period of 10 days, in which the females remained in the males’ cage throughout the period. Twice a day, the presence of a vaginal plug was checked. If positive, the female was separated and weighed. If negative, a vaginal lavage was performed to check for the presence or absence of sperm and to monitor the females’ estrous cycle.

The pregnant females had their weight monitored during gestation, and on P18, a cesarean section was performed, and the total number of reabsorptions, and the total number and weight of fetuses, as well as their respective placentas, were evaluated.

### 2.9. Tissue Processing for Cytokine Quantification and Histological Analysis

To perform cytokine quantification, a lung lobe was homogenized with Turrax in a solution containing protease inhibitor (Roche, San Francisco, CA, USA), 0.1% triton X and PBS; centrifuged at 10,000 rpm for 10 min; and the supernatant was collected and stored at −80 °C for later measurement.

For histological analysis, a fragment of the lung, placenta and testicles were separated for fixation with 4% paraformaldehyde (PFA) and then immersed in 100% alcohol for 40 min to dehydrate the tissue, followed by immersion in xylene for 20 min to clear the tissue. As a final step, the tissues were immersed in paraffin for 50 min, embedded, sectioned and stained with hematoxylin and eosin (HE) for histological analysis.

### 2.10. Histopathological Evaluation of the Lung, Testicle and Placenta

Semiquantitative evaluations were performed under an optical microscope, with complete scanning of the histological section, performed by two pathologists without prior knowledge of the identification of each sample. The presence and type of degenerative phenomena and histological lesions were evaluated. The histopathological score was constructed by searching for common lesions in the lung, such as degeneration; hyperemia/congestion; mono- and polymorphonuclear inflammatory infiltrate in the alveolar and septal spaces; edema; thickening of the alveolar septa; and alveolar, perivascular, peribranchial and pleural infiltrate, hemorrhage, atelectasis, fibrosis and necrosis. The most frequent findings were mono- and polymorphonuclear inflammatory infiltrate in the alveolar and septal spaces, thickening of the alveolar septa and alveolar and peribranchial infiltrate.

For histopathological evaluation of the testicles, the lesions investigated were related to orchitis, edema, cellular hypotrophy, cellular hypertrophy, fibrosis and necrosis. For analysis of the placentas, the histopathological score was constructed by searching for common lesions in the placenta, such as villous immaturity, villitis, thrombosis, calcifications, giant cells, inflammatory infiltrate, edema, hemorrhage, fibrosis, necrosis and deposition of fibroid material.

For all alterations found, it was observed whether these were absent (score 0); discrete, with up to 25% of the sample being affected (score 1); moderate, when 25% to 50% of the sample presented the lesion (score 2) or 50% to 75% of the sample presented the alteration (score 3); and intense when more than 75% of the sample presented the alteration (score 4).

### 2.11. Cytokine Assay

Cytokine quantification was performed using a specific ELISA kit, the BD OptEIATM Set mouse ELISA, for tumor necrosis factor (TNF), interferon gamma (IFN-γ) and interleukins (IL-6). Concentrations were determined according to the manufacturer’s recommendations (B&D Biosciences, Franklin Lakes, NJ, USA).

### 2.12. Quantification of Total Proteins

The colorimetric method with bicinchoninic acid (BCA) was used. The dosage was performed using the commercial protein quantification kit BCATM Protein Assay (ThermoFisher Scientific, Waltham, MA, USA).

### 2.13. RT-qPCR Assay

Approximately 50 mg of lung tissue samples was dissociated using an Ultra-turrax homogenizer, and total RNA was extracted with Trizol reagent (Sigma, Ronkonkoma, NY, USA), following the manufacturer’s instructions. The mRNA concentration was standardized for all samples (50 ng/μL), and quantification of angiotensin-converting enzyme 2 (ECA-2) was performed using the Applied Biosystems TaqPath system (ThermoFisher Scientific, Waltham, MA, USA).

### 2.14. Statistical Analysis

The results are presented as mean ± standard deviation (SD). Comparisons between two independent groups were performed using the nonparametric Mann–Whitney U test. Differences were considered statistically significant when *p* < 0.05. The exact sample size for each experiment is indicated in the corresponding figure legends.

## 3. Results

### 3.1. Instillation with the iSARS-CoV-2 Virus During the Implantation Period Demonstrated Negative Outcomes at the Maternal–Fetal Interface

**Figure 1:**
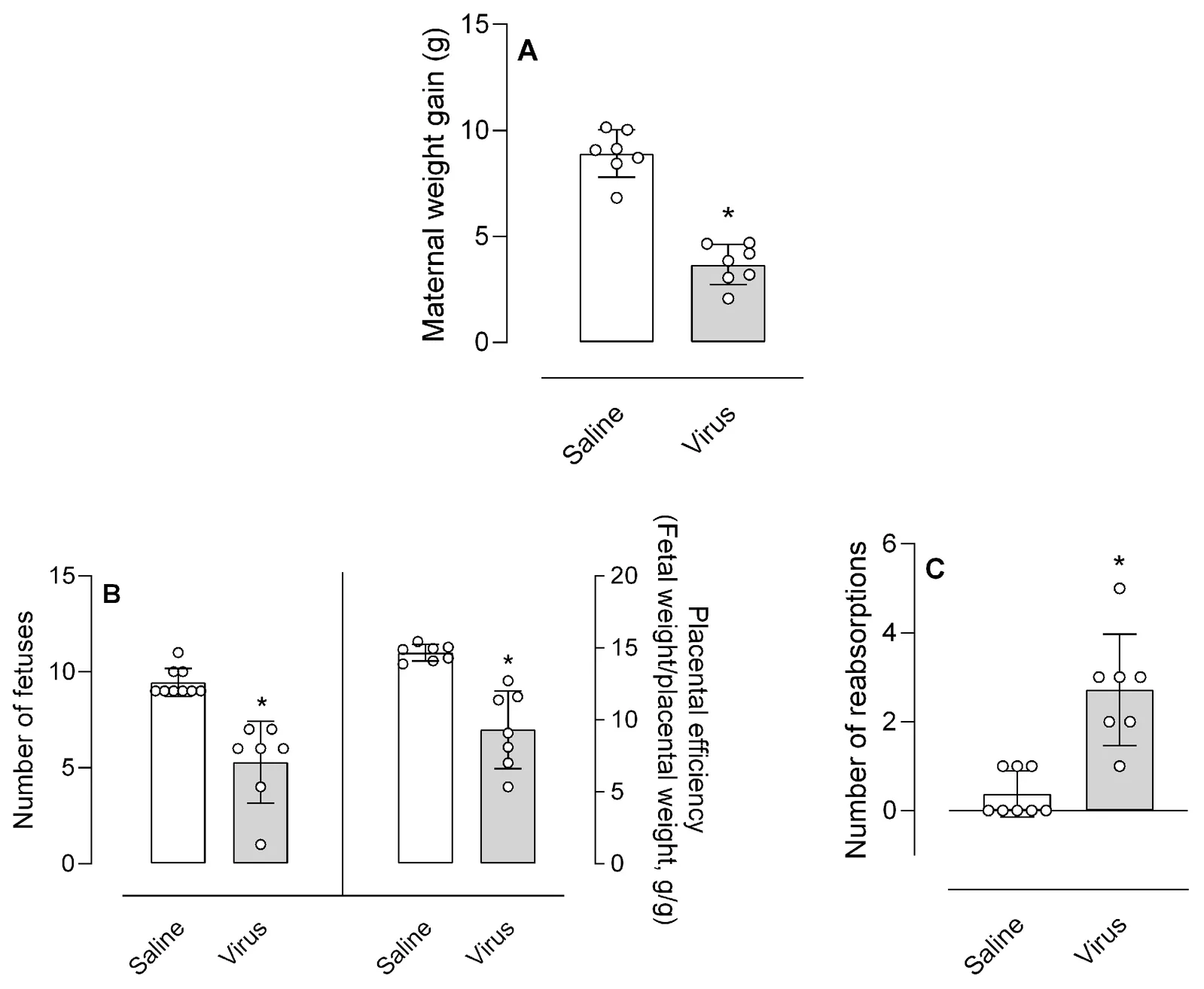
Negative perinatal outcomes in K18-hACE2 mice after 7 days of instillation with iSARS-CoV-2 virus or saline. After the presence of the vaginal plug, the animals were instilled with 5 μL/nostril of saline or 1 × 10^5^ PFU of iSARS-CoV-2 virus during the first 7 days of gestation. On P18, a cesarean section was performed, and the following were analyzed: (**A**) maternal toxicity; (**B**) total number of fetuses (**left**) and placental efficiency (**right**); (**C**) number of resorptions. The results are presented as mean ± standard deviation (Saline group: *n* = 6–9; Virus group: *n* = 7, Each dot represent an individual mouse). Statistical analysis was performed using the Mann–Whitney U test. * *p* < 0.05 compared with saline-instilled mice.

Figure 1A shows that females instilled with iSARS-CoV-2 exhibited a 58.7% reduction in maternal weight gain when compared with females instilled with saline. Figure 1B presents placental efficiency (PE) and the number of live fetuses, demonstrating that viral instillation resulted in both reduced placental efficiency and fewer viable fetuses when compared with fetuses from saline-instilled females. The reduction in PE suggests that placental dysfunction not only impaired fetal growth but also compromised embryonic viability and likely contributed to early embryonic loss, as evidenced by the increased number of reabsorptions observed in virus-instilled females relative to the saline group (Figure 1C).

### 3.2. Exacerbation of the Inflammatory Response After K18-hACE2 Females Instilled with iSARS-CoV-2 During the Embryonic Implantation Period

To assess whether instillation with iSARS-CoV-2 could activate an inflammatory response, on P18, lung fragments were removed for histological analysis and quantification of specific inflammatory cytokines.

**Figure 2:**
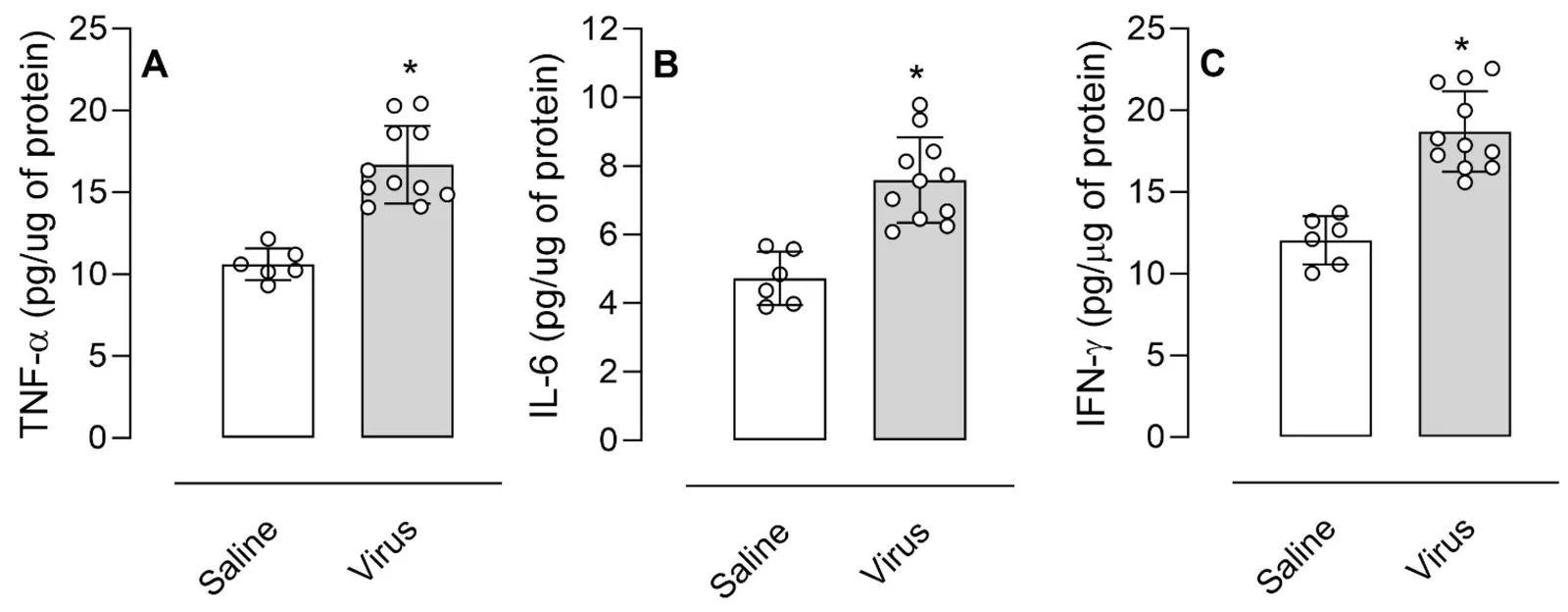
Quantification of TNF-α, IL-6, and IFN-γ in lung homogenates from pregnant K18-hACE2 mice instilled with iSARS-CoV-2 virus or saline. After the presence of the vaginal plug, the animals were instilled with 5 μL/nostril of saline or 1 × 10^5^ PFU of virus during the first 7 days of gestation. On P18, lung fragments were collected for quantification of specific cytokines. (**A**) TNF-α; (**B**) IL-6; (**C**) IFN-γ. The results are expressed as mean ± SD (Saline group: *n* = 6; Virus group: *n* = 11, each dot represent an individual mouse) and were corrected for tissue protein quantification. Statistical analysis was performed using the Mann–Whitney U test. * *p* < 0.05 compared with saline-instilled mice.

Instillation of iSARS-CoV-2 virus resulted in a 35%, 50.4%, and 45.8% increase in the production of tumor necrosis factor (TNF-α), interleukin-6 (IL-6), and interferon-γ (IFN-γ), respectively, when compared with the saline group (Figure 2).

### 3.3. Females Instilled with the iSARS-CoV-2 Virus During the First Seven Days of Gestation Present Significant Histological Changes in Lung Tissue

**Figure 3:**
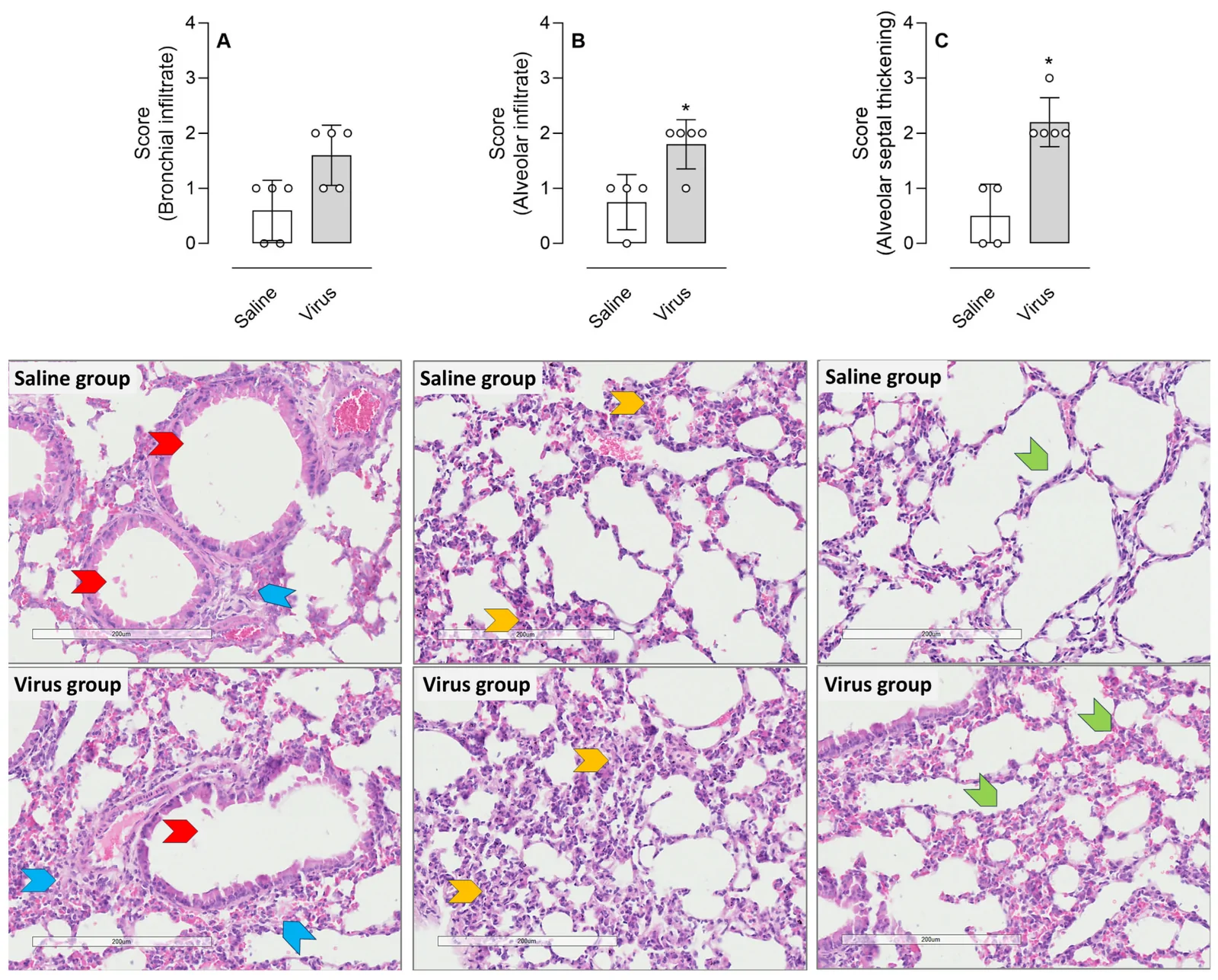
Histopathological analysis and scoring of lung damage in pregnant K18-hACE2 mice instilled with virus or saline. After the presence of the vaginal plug, the animals were instilled with 5 μL/nostril of saline or 1 × 10^5^ PFU of iSARS-CoV-2 during the first 7 days of gestation. On P18, a lung fragment was collected for histopathological analysis. (**A**) Bronchial infiltrate; (**B**) alveolar infiltrate; and (**C**) alveolar septal thickening, where a score related to the degree of biopsy involvement was assigned. Images are representative of each condition. Images were obtained at ×20 magnification, and the arrows indicate bronchi (red), bronchial infiltrate (blue), alveolar infiltrate (yellow) and alveolar septal (green). The results are expressed as mean ± SD (Saline group: *n* = 4–5; Virus group: *n* = 5, each dot represent an individual mouse). Statistical analysis was performed using the Mann–Whitney U test. * *p* < 0.05 compared with saline-instilled mice.

Our results suggest that pregnant mice exposed to iSARS-CoV-2 virus could develop pneumonia. To investigate this hypothesis, lung tissue was collected for histopathological analysis at P18. Figure 3B,C show a significant increase in inflammatory infiltrate and thickening of alveolar septa in iSARS-CoV-2 virus-instilled mice. It is possible to note a tendency for an increase in bronchial inflammatory infiltrate (Figure 3A). It is worth noting that the accumulation of collagen and the presence of inflammatory cells in the septa hinder gas exchange, thus favoring the development of viral-like pneumonia.

### 3.4. Females Instilled with the iSARS-CoV-2 Virus During the Embryonic Implantation Period Did Not Present Histological Changes in the Placenta

**Figure 4:**
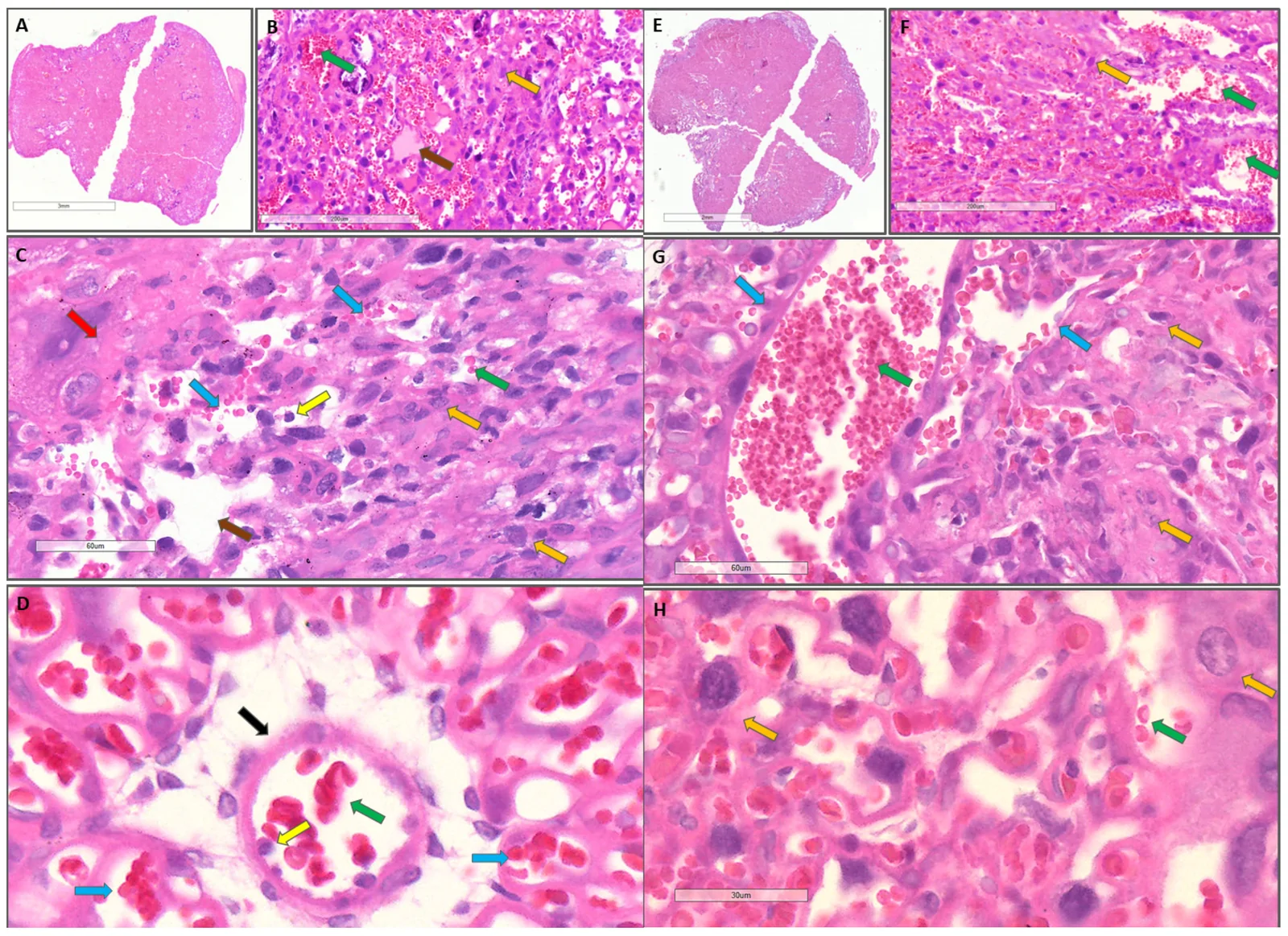
Representative photomicrographs of placentas collected on gestational day 18 (GD18) from pregnant mice intranasally instilled with saline or iSARS-CoV-2 during the implantation period (GD1–GD7). Representative images from the Control group are shown in (**A**–**D**), and those from the iSARS-CoV-2 group in (**E**–**H**). (**A**,**E**) Panoramic views of placental samples (1× magnification; scale bar = 3 mm). (**B**,**F**) Higher-magnification images showing red blood cells (green arrows) and stromal cells (orange arrows); chorionic villi (brown arrow) are also indicated in the Control group (**B**) (20× magnification; scale bar = 200 μm). (**C**,**G**) Decidua (red arrows), chorionic villi (brown arrows), venous capillaries (blue arrows), red blood cells (green arrows), stromal cells (orange arrows), and white blood cells (yellow arrows) (40× magnification; scale bar = 60 μm). (**D**,**H**) Venous capillaries (blue arrows), arterial capillaries (black arrows), red blood cells (green arrows), stromal cells (orange arrows), and white blood cells (yellow arrows) (80× magnification; scale bar = 30 μm). H&E staining.

Due to the low placental efficiency presented by the fetuses of mice instilled with iSARS-CoV-2 virus during the embryonic implantation period, on P18, it was decided to evaluate the morphology of the placentas in search of degenerative changes or histological lesions. During the biopsy analysis, no histological changes were observed in the animals instilled with the virus or with saline (Figure 4).

### 3.5. Sperm Changes in and Reproductive Performance of K18-hACE2 Males After Instillation with the iSARS-CoV-2 Virus

To determine whether the iSARS-CoV-2 virus could interfere with the reproductive parameters of male K18-hACE2 animals, 5 μL/nostril of 1 × 10^5^ PFU of iSARS-CoV-2 virus or 0.9% saline was instilled for 14 consecutive days (spermatogenic cycle time). The animals were then euthanized for collection, weighing, and histological analysis of the testicles, as well as removal of the epididymis for total sperm count and classification. The sperm were classified according to their motility and morphology.

**Figure 5:**
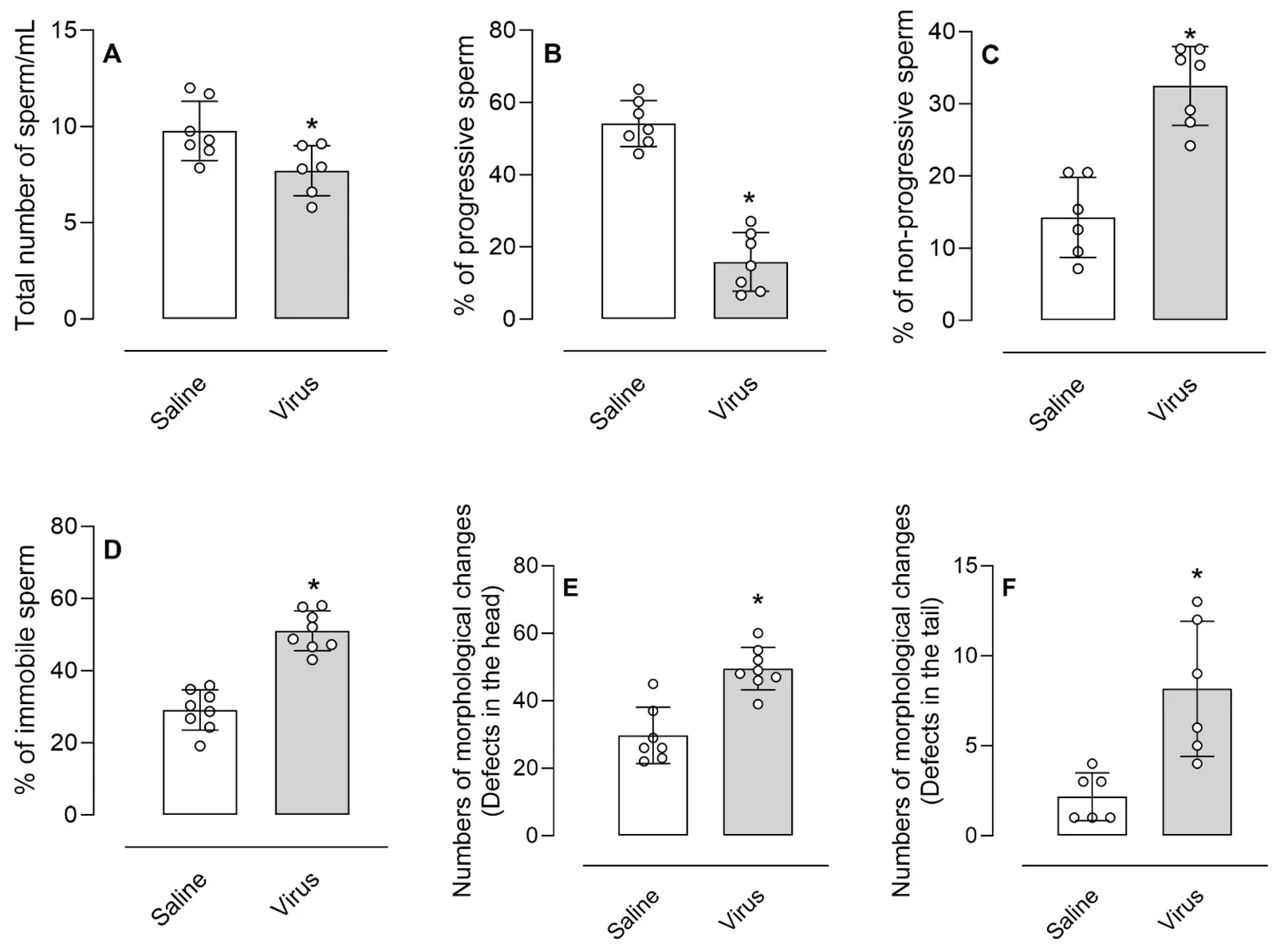
Effects of iSARS-CoV-2 instillation on sperm parameters and morphology in K18-hACE2 mice. Male mice were instilled intranasally with 5 μL/nostril containing 1 × 10^5^ PFU of iSARS-CoV-2 or saline for 14 consecutive days. At the end of the experimental period, animals were euthanized, and the epididymis and vas deferens were bilaterally collected and incubated in an appropriate buffer to allow sperm migration. (**A**) Total sperm count; (**B**) percentage of progressive sperm; (**C**) percentage of non-progressive sperm; (**D**) percentage of non-motile sperm. For morphological analysis, sperm samples were smeared onto glass slides, stained using a Panoptic staining kit, and analyzed under a light microscope at 100× magnification. (**E**) Sperm head defects; (**F**) sperm tail defects. The results are presented as mean ± standard deviation (Saline group: *n* = 6–8; Virus group: *n* = 6–8, each dot represent an individual mouse). Statistical analysis was performed using the Mann–Whitney U test. * *p* < 0.05 compared with saline-instilled mice.

However, it is possible to observe in Figure 5A a significant reduction of 18% in the total number of sperm in the iSARS-CoV-2 virus-instilled group. Surprisingly, it was also observed that there was a significant change in the motility classification of these sperm. A percentage relationship was established between each classification (A + B, C or D) with the total number of sperm. Thus, Figure 5B shows that there was a significant reduction in the percentage of progressive sperm (A + B) of 70.5% and, consequently, an increase in the percentage and number of non-progressive and immobile sperm of 127% and 76%, respectively (Figure 5C,D).

The morphological analysis of the spermatozoa was performed according to the criteria stipulated by [28], considering alterations/defects found in the head and tail of the spermatozoa. Figure 5E,F show that the animals instilled with the virus presented a significantly greater number of alterations, both in the head and in the tail, when compared to the group of animals instilled with saline.

Due to the alterations observed in sperm parameters, particularly those related to motility and morphology, the question arose as to whether these changes could affect the reproductive performance of the animals, specifically, their ability to fertilize healthy, nulliparous females. Therefore, after 14 consecutive days of viral instillation, male mice (saline, *n* = 7; iSARS-CoV-2, *n* = 7) were each housed individually with two nulliparous females for a mating period of 10 days, and mating success was defined as detection of vaginal plugs in both females. It was observed that males instilled with iSARS-CoV-2 exhibited evidence of mating, as assessed by vaginal plug detection, only after 96 h of direct contact with females. In addition, 28.6% of virus-instilled males did not mate with the females during the 10-day experimental period. In contrast, saline-instilled mice showed 100% mating success within less than 48 h (Table 1).

### 3.6. Changes in the Number of Offspring Are Observed in Females That Mated with Males Instilled with the iSARS-CoV-2 Virus for 14 Consecutive Days

**Figure 6:**
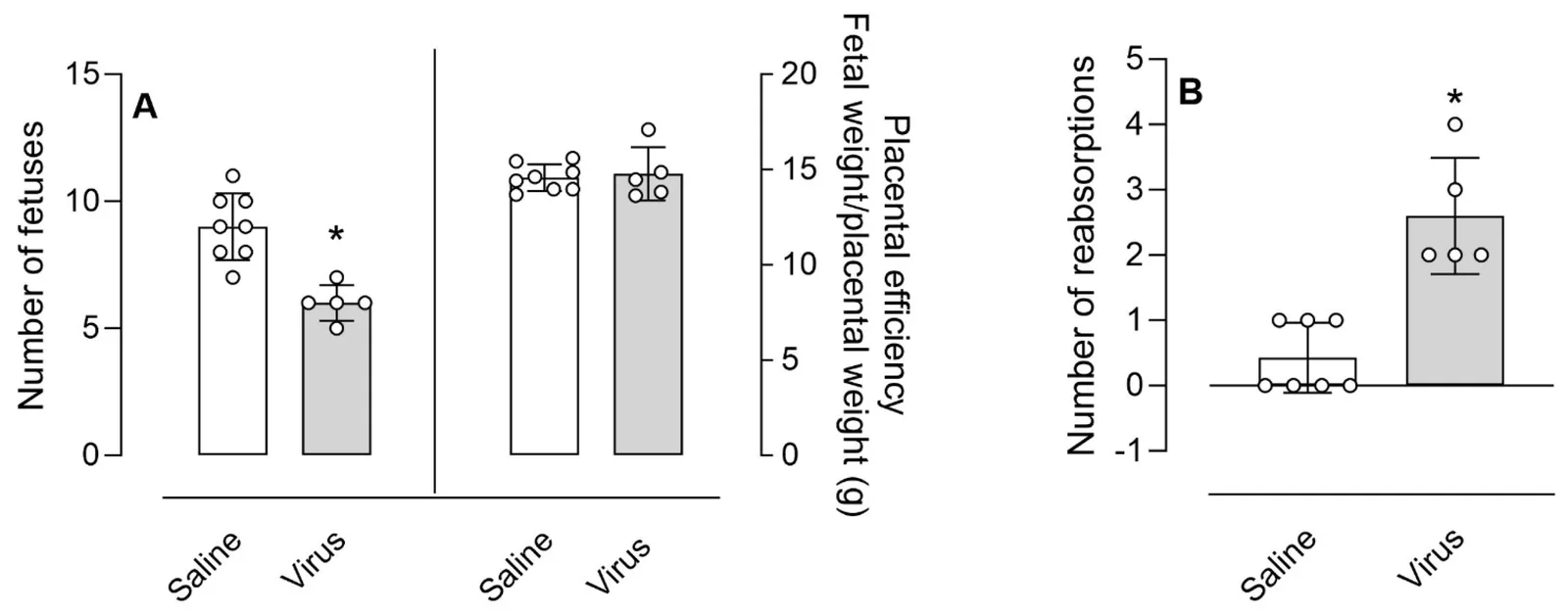
Change in the number of fetuses in female mice that mated with males instilled with virus or saline for 14 days. After 14 consecutive days of instillation with 5 μL/nostril of saline or 1 × 10^5^ PFU of iSARS-CoV-2, males were mated with healthy, nulliparous females for 10 days. On P18, a cesarean section was performed. (**A**) Total number of fetuses (**left**) and placental efficiency (**right**); (**B**) number of resorptions. The results are presented as mean ± standard deviation (Saline group: *n* = 7–8; Virus group: *n* = 5, each dot represent an individual mouse). Statistical analysis was performed using the Mann–Whitney U test. * *p* < 0.05 compared with saline-instilled mice.

Females mated with virus-instilled males exhibited a 28.8% reduction in the number of fetuses, without significant changes in placental efficiency between groups, indicating that these events occurred independently (Figure 6A). In contrast, a significant increase in the number of reabsorptions was observed in females mated with virus-instilled males when compared with the saline group (Figure 6B), indicating increased embryonic loss, whereas placental efficiency remained unchanged among the surviving fetuses.

### 3.7. Inflammatory Response Triggering and Lung Damage Are Observed in Male K18-hACE2 Mice After 14 Days of Instillation with the iSARS-CoV-2 Virus

**Figure 7:**
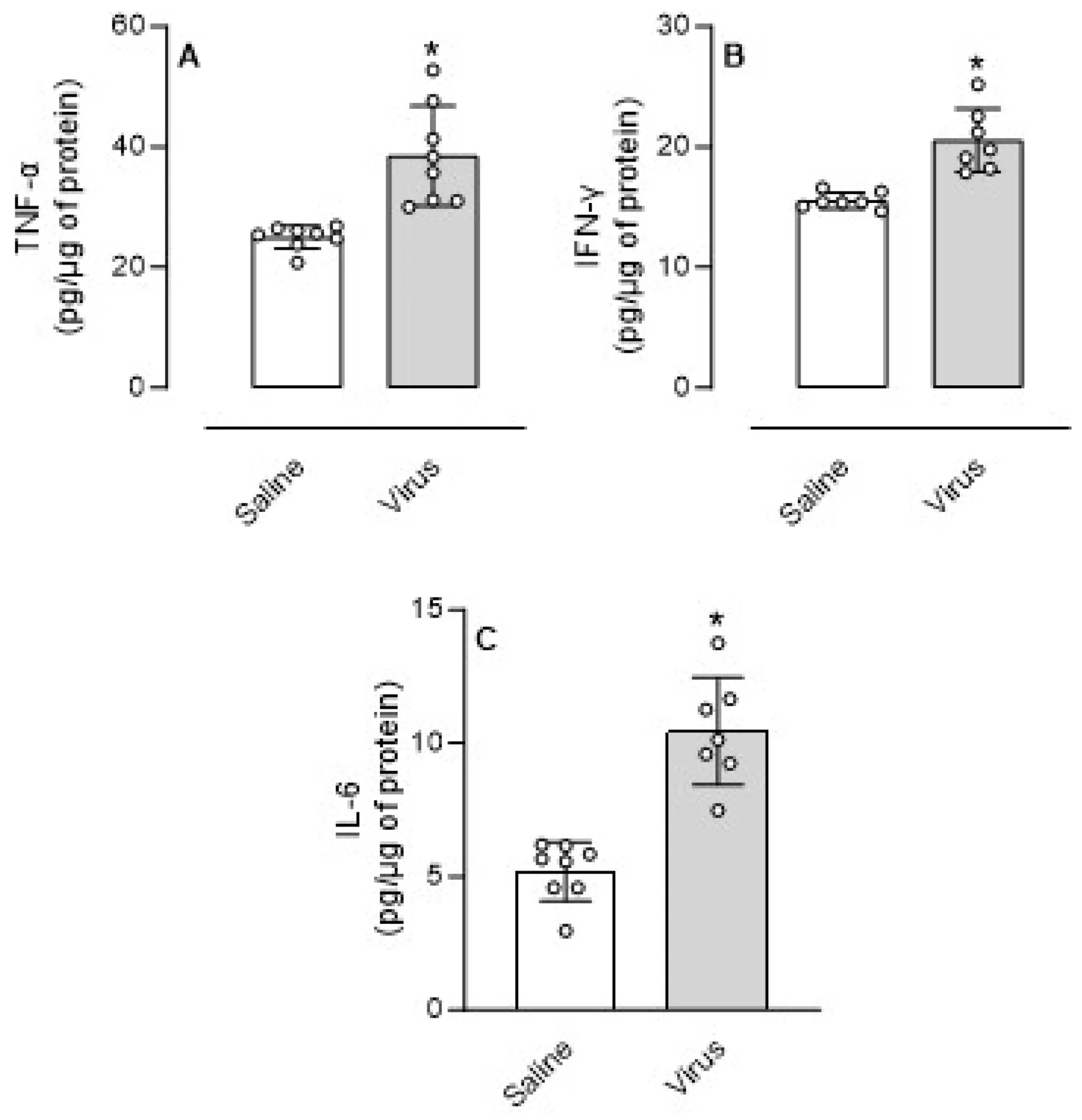
Quantification of TNF-α, IL-6, and IFN-γ cytokines in lung homogenates from male K18-hACE2 mice instilled with iSARS-CoV-2 or saline. Male mice were instilled for 14 consecutive days with 5 μL/nostril of saline or 1 × 10^5^ PFU of virus. On the day of euthanasia, lung fragments were collected for quantification of specific cytokines. (**A**) TNF-α graph, (**B**) IFN-γ, (**C**) IL-6. The results are presented as mean ± standard deviation (Saline group: *n* = 7–8; Virus group: *n* = 7–8, each dot represent an individual mouse). Statistical analysis was performed using the Mann–Whitney U test. * *p* < 0.05 compared with saline-instilled mice.

We next investigated whether an inflammatory process would develop in males instilled with the virus for 14 consecutive days. Virus-instilled males showed increased pulmonary levels of TNF-α, IFN-γ and IL-6, which were elevated by 54%, 32.3% and 100%, respectively, relative to saline-instilled controls (Figure 7A–C).

**Figure 8:**
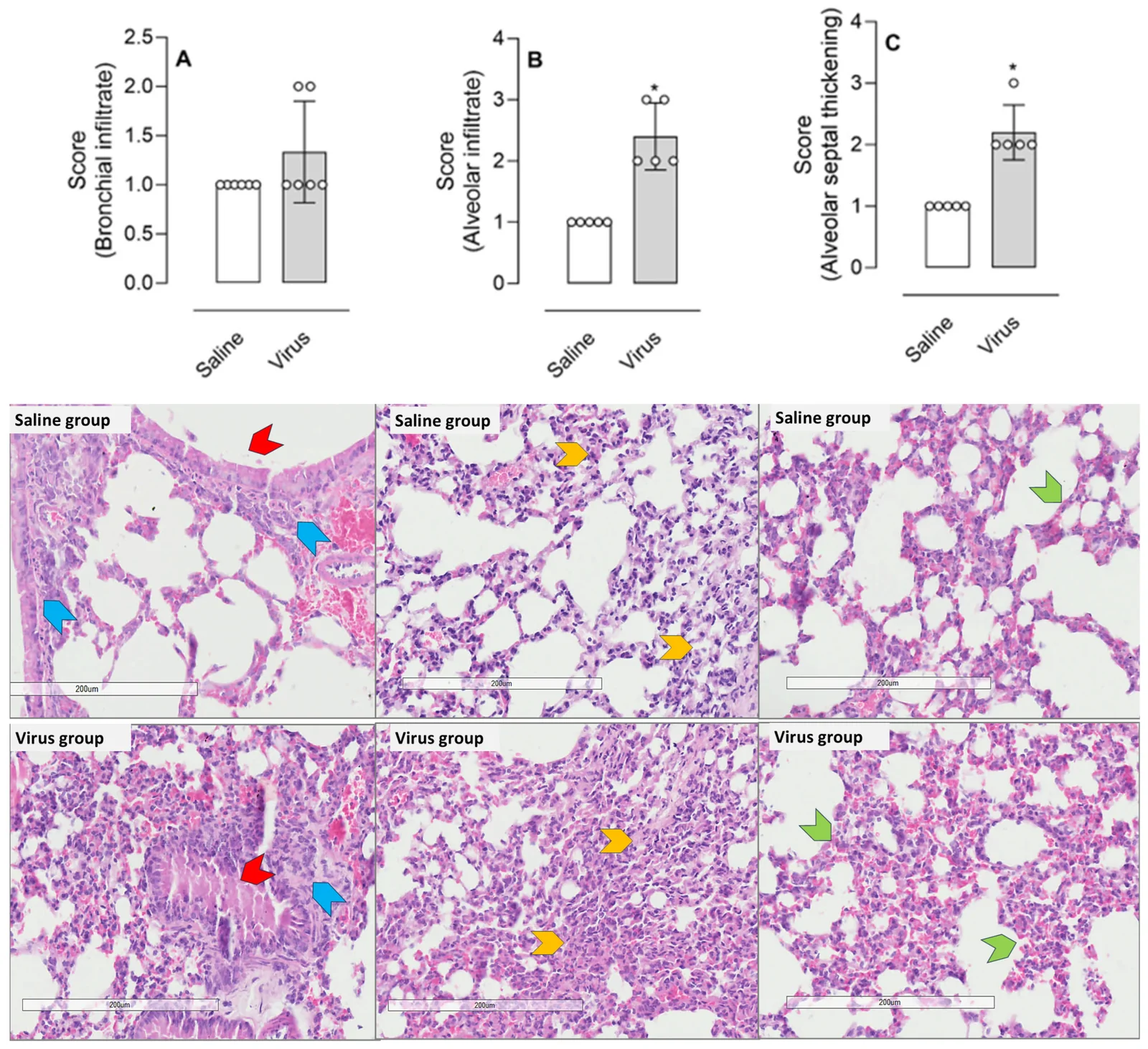
Histopathological analysis and scoring of lung damage in male mice instilled with iSARS-CoV-2 or saline. Male mice were instilled for 14 consecutive days with 5 μL/nostril of saline or 1 × 10^5^ PFU of iSARS-CoV-2 virus. On the day of euthanasia, a lung fragment was collected for histopathological analysis, and the following parameters were observed. (**A**) Bronchial inflammatory infiltrate, (**B**) alveolar inflammatory infiltrate, and (**C**) thickening of the alveolar septa, where a score related to the degree of biopsy involvement was assigned. Histopathological analyses were done at ×20 magnification, and the arrows indicate bronchi (red), bronchial infiltrate (blue), alveolar infiltrate (yellow) and alveolar septa (green). The results are presented as mean ± standard deviation (Saline group: *n* = 5–6; Virus group: *n* = 5–6, each dot represent an individual mouse). Statistical analysis was performed using the Mann–Whitney U test. * *p* < 0.05 compared with saline-instilled mice.

The analysis of lung biopsies showed that mice instilled with the virus exhibited a trend toward increased lung damage related to bronchial alterations, along with a significant increase in alveolar inflammatory infiltrate and thickening of the alveolar septa (Figure 8A–C).

## 4. Discussion

In the present study, we established a transgenic mouse model of iSARS-CoV-2 instillation during pregnancy that reproduces several clinical features of COVID-19 in pregnant women. K18-hACE2 females exposed during embryonic implantation showed significant reproductive impairment, including fewer viable fetuses; increased embryonic resorptions; and maternal toxicity, evidenced by low or absent gestational weight gain. Moreover, 66% of females exhibited reduced placental efficiency, suggesting impaired placental function.

Recent studies indicate that SARS-CoV-2 infection during pregnancy can impair fetal development, largely through maternal inflammatory responses [29,30,31]. In pregnant CD1 mice, mouse-adapted SARS-CoV-2 (maSCV2) increased maternal morbidity and induced fetal growth restriction and trophoblast loss, associated with placental inflammation [13]. Similarly, gestational SARS-CoV-2 exposure in K18-hACE2 mice caused persistent neurobehavioral deficits in offspring, associated with maternal immune activation and inflammation in placental and fetal compartments [32]. Increased cytokine production may alter the placental barrier and affect fetal development [33], consistent with evidence from human studies [10,11].

Our findings suggest that iSARS-CoV-2 induces a strong inflammatory response that disrupts the uterine environment and maternal–fetal interface, even in the absence of viral replication. Pregnant females also exhibited pulmonary inflammation, characterized by increased inflammatory cytokines, alveolar infiltration, and septal thickening. These findings agree with studies showing that SARS-CoV-2 or its Spike S1 subunit induces cytokine production, lung injury, increased vascular permeability, and impaired epithelial barrier function in K18-hACE2 mice [33,34]. Thus, viral structural components may activate innate immunity independently of replication [35,36,37].

Multiple, non-mutually exclusive pathways could account for the inflammatory response observed. Viral structural components retained in the inactivated preparation are recognized by innate immune sensors: the envelope protein is sensed by TLR2 independently of viral entry [38], and evidence indicates that the Spike protein may bind TLR4 with high affinity, potentially contributing to COVID-19-associated hyperinflammation [39,40,41]. TLR4 activation induces MyD88- and TRIF-dependent pathways, promoting production of IL-6, TNF-α, IL-1β, and inflammatory chemokines [39,42], and dysregulated TLR4 activation has been implicated in COVID-19 pathogenesis and severe manifestations, including lung injury, ARDS, thromboembolic events, and multiple organ failure [43,44]. In parallel, Spike engagement of hACE2 can exert biological effects independently of productive viral replication, including ACE2 downregulation and disruption of local renin–angiotensin homeostasis [45]. None of these pathways was directly assessed in the present study, and their relative contribution to the maternal–fetal and pulmonary inflammatory responses reported here remain to be experimentally defined.

Because exposure to iSARS-CoV-2 impaired pregnancy and induced pulmonary inflammation in females, we also investigated reproductive effects in K18-hACE2 males. Virus-exposed males showed reduced total and progressive sperm counts, increased non-progressive or immotile sperm, and increased morphological abnormalities, particularly in the sperm head and tail. In contrast, testicular histology was unchanged, suggesting that these effects may reflect impaired spermatogenesis or sperm function rather than major structural testicular damage.

Inflammation, cytokine dysregulation, and oxidative stress are recognized mechanisms of SARS-CoV-2-associated male reproductive impairment [46,47,48]. In K18-hACE2 mice, Giannakopoulos et al. [20] identified cytokine storm as a major contributor to testicular injury, reduced sperm count, and impaired fertility. Similarly, Martinez et al. [21] reported seminal inflammation and reduced sperm parameters in men recovering from COVID-19, although these alterations were transient and recovered three months after disease resolution.

Consistent with the sperm abnormalities, males exposed to iSARS-CoV-2 showed reduced reproductive efficiency. Mating was delayed, with approximately 72 h required to obtain a positive vaginal plug compared with 48 h in controls, and only 71% of exposed males successfully mated versus 100% of controls. Among successful matings, females paired with exposed males exhibited fewer fetuses and increased embryonic resorptions, although placental efficiency was unchanged. These findings suggest that virus-associated sperm dysfunction may compromise embryo viability and pregnancy outcome. Oxidative stress, sperm DNA fragmentation, and morphological abnormalities have been associated with impaired embryonic development, reduced embryo quality, aneuploidy, and pregnancy failure [49,50].

Male exposure also induced an exacerbated pulmonary inflammatory response, consistent with previous reports [51,52]. These findings indicate that inactivated viral components are sufficient to activate respiratory immune responses in this model and may contribute to the systemic and reproductive alterations observed.

A question that follows from these observations is whether the inflammatory response described here requires Spike–hACE2 engagement, or whether wild-type mice would have responded similarly through recognition of pathogen-associated molecular patterns (PAMPs). These possibilities are not mutually exclusive. As discussed above, inactivated whole-virus preparations retain multiple PAMPs capable of activating innate signaling independently of ACE2, and these pathways are fully preserved in non-transgenic animals. Receptor engagement, however, provides an additional and genotype-restricted arm: murine ACE2 binds the ancestral Spike poorly, Spike-driven ACE2 downregulation with local renin–angiotensin imbalance occurs in the absence of viral replication [27], and the same non-replicating Spike-derived stimulus that induces lung injury in K18-hACE2 mice [34] produces a markedly milder phenotype in wild-type animals [26]. This distinction is particularly relevant to the reproductive endpoints reported here, given that ACE2 is expressed at the maternal–fetal interface and in gonadal tissue, where it participates in local vascular and steroidogenic regulation. Our design cannot formally discriminate between these mechanisms, as non-transgenic controls and receptor blockade were not included. The present data therefore indicate that exposure to inactivated SARS-CoV-2 is sufficient to elicit inflammation and adverse reproductive outcomes in a receptor-humanized host, but do not establish that the Spike–hACE2 interaction is necessary for these effects. Parallel exposure of wild-type C57BL/6 and K18-hACE2 animals, combined with ACE2 blockade and TLR2/TLR4 inhibition, will be required to resolve this question and represents a necessary direction for future studies.

Taken together, our findings demonstrate that iSARS-CoV-2 exposure compromises reproductive function in both K18-hACE2 females and males. In females, exposure reduced fetal viability, increased embryonic resorption, impaired placental efficiency, and induced pulmonary inflammation. In males, it reduced sperm quality and reproductive efficiency and negatively affected pregnancy outcomes in mated females. These effects were accompanied by increased inflammatory cytokines and pulmonary histopathological changes, indicating that exposure to non-replicating viral components is sufficient to compromise reproductive outcomes in a receptor-humanized host. Whether these effects arise from systemic inflammation, from local response in ACE2-expressing reproductive tissue, or from both remains to be determined.

## 5. Conclusions

Taken together, our results demonstrate that exposure to the iSARS-CoV-2 virus compromises reproduction in both K18-hACE2 females and males. In pregnant females, it caused a reduction in the number of fetuses, increased resorptions, and exacerbated lung inflammation, while in males, it resulted in decreased sperm quality, lower reproductive efficiency, and negative outcomes for the gestational viability of mated females.

## Figures and Tables

**Table 1:** Daily mating success of male K18-hACE2 mice instilled with iSARS-CoV-2 or saline. Males were instilled for 14 consecutive days with 5 μL/nostril of saline or 1 × 10^5^ PFU of iSARS-CoV-2 and subsequently housed individually with two nulliparous females for 10 days. Each male remained in the experiment until vaginal plugs were detected in both females, and it was recorded the day on which mating with the second female was confirmed. Values indicate the percentage of males in each group completing mating on the corresponding day, with the number of males over the group total shown in parentheses. Saline group: *n* = 7; iSARS-CoV-2 group: *n* = 7.

Experimental Group	Day 1	Day 2	Day 3	Day 4	Day 5	Days 6–10	Did Not Mate
Saline (*n* = 7)	57% (4/7)	43% (3/7)	0	0	0	0	0
iSARS-CoV-2 (*n* = 7)	0	0	0	43% (3/7)	28.6% (2/7)	0	28.6% (2/7)

## Data Availability

The data will be made available upon reasonable request.
